# Benign tumors and non-melanoma skin cancers in patients with Fanconi anemia

**DOI:** 10.1007/s10689-024-00410-2

**Published:** 2024-06-21

**Authors:** Aura Enache, Bia Sajjad, Burak Altintas, Neelam Giri, Lisa J. McReynolds, Edward W. Cowen

**Affiliations:** Division of Cancer Epidemiology and Genetics, Clinical Genetics Branch, National Cancer Institute, 9609 Medical Center Drive 6E434, Bethesda, MD 20892 USA

**Keywords:** Fanconi anemia, Basal cell carcinoma, Squamous cell carcinoma, Skin cancer, Benign tumor

## Abstract

Fanconi anemia (FA) is an inherited bone marrow failure syndrome (IBMFS) characterized by pathogenic variants in the FA/BRCA DNA repair pathway genes. Individuals with FA have an elevated risk of developing myelodysplastic syndrome, acute myeloid leukemia, and solid tumors. Hematopoietic cell transplantation (HCT) is the most effective treatment for FA related bone marrow failure but can increase the risk of cancer development. Information on benign tumors and NMSC is lacking in patients with FA. Our objective was to characterize patients with FA enrolled in the National Cancer Institute IBMFS Study who have experienced non-melanoma skin cancers (NMSC) and/or benign tumors (BT). A total of 200 patients diagnosed with FA were enrolled in the Institutional Review Board approved study “Etiologic Investigation of Cancer Susceptibility in IBMFS: A Natural History Study” (NCT00027274). Through medical records review, we identified 30 patients with at least one NMSC, either squamous or basal cell carcinoma, or benign tumor. The remaining 170 patients comprised the control group. Out of 200 patients, 12 had NMSC, 25 had benign tumors, with an age range of 11–64 and 0–56 years, respectively. The median age at HCT was 30.5 years for NMSC patients, 9 years for benign tumor patients, and 9.1 years for controls. The most common genotype observed was *FANCA*, followed by *FANCC* and *FANCI*. Benign tumors spanned diverse anatomical locations. Early onset NMSC in patients with FA compared to the general population emphasizes the need for consistent monitoring in patients with FA, while the diverse anatomical locations of benign tumors underscore the importance of comprehensive surveillance for timely interventions in managing symptomatology and heightened cancer risk.

**Supplementary Information:**

The online version contains supplementary material available at 10.1007/s10689-024-00410-2.

## Introduction

Fanconi anemia (FA) is a rare genetic disorder that is generally characterized by physical abnormalities, inherited bone marrow failure (BMF), and an increased susceptibility to malignancies [1, 2]. FA is caused by pathogenic variants in the genes making up the FA/BRCA DNA repair pathway, which consists of 22 proteins, organized into complementation groups based on their position in the FA pathway [3]. Inheritance patterns for most FA genes are biallelic, autosomal recessive, except *FANCB* which is X-linked and *FANCR* (*RAD51*), which is autosomal dominant [4, 5]. Patients with FA exhibit a diverse range of clinical presentations, typically diagnosed around the age of 7 years [1, 6]. These clinical presentations include pancytopenia, skin abnormalities, upper limb abnormalities, and short stature [1]. A common curative treatment for BMF secondary to FA is hematopoietic cell transplantation (HCT). However, the risk of developing cancer is additionally heightened for individuals who undergo transplant [7].

Patients with FA face a heightened risk of developing cancer, particularly hematological disorders such as myelodysplastic syndrome (MDS) and acute myeloid leukemia (AML), with cumulative incidence rates of 40% and 10% respectively by the age of 50 [6, 8]. The cumulative incidence of solid tumors reaches 20–30% by age 50 [6, 8]. Among the solid tumors observed in patients with FA, head and neck squamous cell carcinoma (SCC), esophageal, brain, and anogenital cancers are the most common [9, 10].

We earlier reported cases of skin SCC and basal cell carcinoma (BCC) in patients with FA for cases through 2015 [9, 10]. Our current objective was to characterize patients with FA who are enrolled in the National Cancer Institute (NCI) inherited bone marrow failure syndrome (IBMFS) cohort who have experienced non-melanoma skin cancers (NMSC), either SCC or BCC, and/or benign tumors (BT). We elected to examine both BT and NMSC despite their differences because they are both understudied in this population. Since FA affects most organs of the body, any information that can aid in patient surveillance is valuable. Anecdotal observations suggested an increase in NMSCs and BTs, prompting us to report these findings, and there have been no previous reports on benign tumors (BT) in patients with FA. This case series aims to shed light on the occurrence and clinical features of these tumors in the context of FA. We investigated various factors, such as the age at the first adverse event, HCT status, biological sex, FA complementation group, cumulative incidence, and survival probability.

## Methods

Individuals diagnosed with FA enrolled in the Etiologic Investigation of Cancer Susceptibility in Inherited Bone Marrow Failure Syndromes: A Natural History Study, which was approved by the NCI Institutional Review Board (NCT00027274). Patients were followed from time of enrollment (earliest January 2002) through October 2023. A total of 200 patients with FA were enrolled. There were 143 patients in the field cohort, and 57 in the clinic cohort. Participants or their proxies provided written consent and authorized the release of their medical records. Data were gathered from individual questionnaires, biennial follow-up forms, medical records, and evaluations conducted at the National Institutes of Health Clinical Center (for 28.5% of patients). The diagnosis of FA was made based on an abnormal chromosomal breakage test and was further confirmed through genetic testing when possible.

Retrospective review of medical records identified thirty patients within this study having at least one adverse event of interest: NMSC (SCC, BCC), or benign tumors (BT). Twenty-four of these patients were in the clinic cohort. The diagnoses of SCC, BCC, or BT were reported through various methods, such as pathology reports, surgery/operatiave reports, physician/consultation reports, self-reports, and personal relations reports. Most NMSCs were reported by pathology or surgery/operation report, and approximately 5% were reported by self report or a personal relations report, which is defined as information via a parent or family member. The control group consisted of the remaining 170 FA patients with available data. HCT status, sex distribution, and survival probability were compared between FA patients with an adverse event and the control group. Kaplan-Meier method was used to estimate survival probabilities and cumulative incidence. If a patient had more than one NMSC or BT, data for only the first event of NMSC and/or BT were used to calculate cumulative incidence. The time to the event of interest was measured from the individual’s date of birth to the occurrence of the first NMSC, BT or their last follow-up date, with censoring applied to those who did not experience an adverse event at their respective last follow-up point. For comparisons between groups, the log-rank test was utilized. *P*-values less than 0.05 were considered statistically significant. Events with available data were used for age range, median age, HCT status before an adverse event, cumulative incidence, and survival probability analysis.

One patient with a single event of BT was excluded from age range, median age, HCT status before an adverse event, and cumulative incidence, as information on the age at tumor diagnosis was not provided. Last follow-up age for two patients in the control group was less than one year old, and data for these patients was omitted in survival probability and cumulative incidence analysis. Patients reported alive in October 2023, were assumed alive, and their last follow-up date according was utilized for age calculation. We completed data analysis using GraphPad Prism 8, Venny 2.1, and Microsoft Excel.

## Results

Out of 200 patients with available data, 12 had at least one NMSC and 25 had at least one BT (Table 1). The majority were self-identified as white. Nine patients in the NMSC group and 18 in the BT group had bone marrow failure (BMF) compared to 85 in the control group, with median ages of 16.3, 7.5, and 6.5, respectively (Table 1). The median age at death for males versus females in this group was significantly different, at 15.16 years versus 40.13 years, respectively. However, the small sample size, with only 3 males compared to 9 females, likely skewed this median.

**Table 1:** Patient demographics

	NMSC	BT	Controls
Number of individuals	12	25	170
**Sex**			
Female	8	18	93
Male	4	7	77
**Self-reported race/ethnicity**			
White	10	24	106
Hispanic	1	1	17
Asian		1	10
American Indian/Alaska Native			5
African American			6
Unknown	1		32
**Vital Status**			
Total patients alive	3	15	114
Current median age (range)	44.6 (39.9–67.4)	23.15 (9.2–67.4)	36.3 (24–49)
Total patients deceased	9	10	56
Median age at death (range)	33.3(15–44)	40.13 (17–56)	15.16 (3–33)
**HCT Status**			
Patients transplanted	6	11	85
Median age at transplant (range)	30.5 (8.2–36.6)	9.0 (1.5–44.1)	9.1 (3.8–43.3)
**Physical Phenotype**			
Patients with BMF	9	18	142
Median age at BMF (range)	16.3 (5-32.1)	7.5 (0.3–18.9)	6.5 (0.2–40.7)
Median age at first malignancy	29.8	30.7	28.3
≥ 3 VACTERL-H	2	11	37
≥ 4 PHENOS	5	16	62

HCT: hematopoietic cell transplant

BMF: bone marrow failure

VACTERL-H: vertebral, anal, cardiac anomalies, tracheal-esophageal fistula, esophageal/duodenal atresia, renal, upper limb (radial ray) anomalies, hydrocephalus

PHENOS: skin pigmentation changes, small head, small Eyes, central nervous system, otologic anomalies, short stature

The association between HCT and early malignancy onset in FA patients has been previously documented [10]. To evaluate whether this association exists in our patient cohort, we compared the HCT status of FA patients with NMSC to that of the control group. Of the 12 patients with NMSC, six (50%) underwent transplant, and the median age at transplant for this group was 30.5 years (Table 2). Three patients received transplant prior to their first skin cancer while the other three underwent transplant after their first skin cancer. Five patients were deceased at last follow up while one patient in this group was alive. In the control group, 85 (50.3%) of FA patients had an HCT, with a median age of 9.1 years (range 4 to 43 years).

**Table 2:** Characteristics of patients with non-melanoma skin cancers

	NMSC Patient Cohort	Patients with SCC Occurrences	Patients with BCC Occurrences
**Total**	12	8	7
**First NM-Skin Cancer(s)**			
Median age in years (range)	31.3 (11–64)	35.9 (25–64)	31.0 (11–41)
**Number of NM Skin Cancer(s)**			
1	6	6	3
2	4	1	3
3	1	0	0
> 3	1	1	1
Range of number of events	1–26	1–11	1–15
**Sex**			
Female	8	6	4
Male	4	2	3
**Genotype**			
*FANCA*	9	6	5
*FANCC*	1	1	0
Gene Unknown	2	1	2
**HCT Status**			
Patients transplanted	6		
Median age at transplant	30.5		
HCT prior to first NMSC	3		

SCC: squamous cell carcinoma

BCC: basal cell carcinoma

NM: non-melanoma

HCT: hematopoietic cell transplant

Among the 12 patients identified with NMSC, 8 patients had a diagnosis of SCC, 7 had a diagnosis of BCC, and three patients had both SCC and BCC (Supplemental Fig. 1). The median age for the first occurrence of NMSC was approximately 31.3 years. The median age at first diagnosis were 35.9 years (range 11–64) for SCC and 31.0 years for BCC (range 11–41). Six patients (50%) had one NMSC occurrence, four patients (33%) had two, one patient (8.3%) had three, and one patient (8.3%) had more than three occurrences. The range of events per patient extended from 1 to 26. One patient had 11 events of SCC and 15 events of BCC (Table 2).

Within the NMSC patient group we found that eight (67%) were female cases and four (33%) were male cases (Supplemental Fig. 2). In the SCC subgroup, six (75%) were female and two (25%) were male patients. BCC was identified in four (57%) female patients and three (43%) male patients. Furthermore, eight (42%) out of 19 females experienced at least one event of SCC or BCC, while four (33%) out of 12 males had such events.

Twenty-five individuals developed at least one event of a BT. The median age at first diagnosis of BT in the cohort was 17.5 years (range 0–56). Seventeen (68%) patients experienced one, five (20%) patients had two, two (8%) patients encountered three, and one (4%) patient had more than three instances of BT (Table 3). Three benign tumors were endocrine in origin, six within the central nervous system (CNS), seven were associated with solid organs, eight involved the reproductive system and breast tissue, seven were categorized as cutaneous lesions, and six were grouped as other (Table 4).

**Table 3:** Characteristics of patients with benign tumors

	Total Observed
**Total**	25
**First benign tumor reported**	
Median age in years (range)	17.5 (0–56)
**Number of benign tumors**	
1	17
2	5
3	2
> 3	1
Range of number of events observed/patient	1–4
**Sex**	
Female	18
Male	7
**Anatomical Classification**	
Endocrine System	3
Central Nervous System	6
Solid Organs	7
Reproductive System and Breast Tissue	8
Skin	7
Other	6
**Genotype**	
*FANCA*	16
*FANCC*	2
*FANCD1*	1
*FANCG*	1
*FANCI*	2
*FANCJ*	1
*FANCR*	1
Gene Unknown	2
**HCT Status**	
Patients transplanted	11
Median age in years at transplant	9
HCT prior to first tumor	6

HCT: hematopoietic cell transplant

**Table 4:** Benign tumor classifications

Endocrine System	Central Nervous System	Solid Organs	Reproductive System and Breast Tissue	Cutaneous Lesions	Other
Pituitary adenoma (2)	CNS lipoma (2)	Gallbladder polyp (1)	Uterine fibroid (2)	Hemangioma (3)	Tumor, NOS (2)
Thyroid colloid cyst (1)	Parietal lobe mass (2)	Liver adenoma (1)	Testis cyst (1)	Dysplastic nevi (2)	Benign cyst / granuloma (1)
	Cerebellar venous angioma (1)	Focal nodular hyperplasia (1)	Follicular cyst (1)	Neck dermatofibroma (1)	Aneurysmal bone cyst (1)
	Pineal cyst (1)	Liver hemangioma (1)	Ovarian cyst (1)	Warts (anogenital) (1)	Esophageal polyps (1)
		Simple renal cyst (1)	Adnexal cyst (1)		
		Renal cyst (1)	Breast cyst (1)		
		Kidney nephroblastic proliferation (1)	Breast tubular adenoma (1)		

Benign tumors occurred across various anatomical regions. In the endocrine system, we identified an instance of a thyroid cyst and two adenomas in the pituitary gland. In the CNS, two cases of lipoma were identified, with one located in the third ventricle, and the other reported as a spinal lipoma. Additionally, we identified two cases of a parietal lobe mass (histopathology unknown), one case of cerebellum angioma, and one case of a pineal region cyst. Occurrences involving the gallbladder, liver, kidney, and lung were classified as solid organ benign tumors. In the reproductive system and breast tissue, BT instances included cysts of the testis, ovaries, uterus, as well as breast tubular adenoma and uterine fibroids. The cutaneous lesions category included anal warts, hemangiomas, dysplastic nevi, and dermatofibromas.

**Fig. 1:**
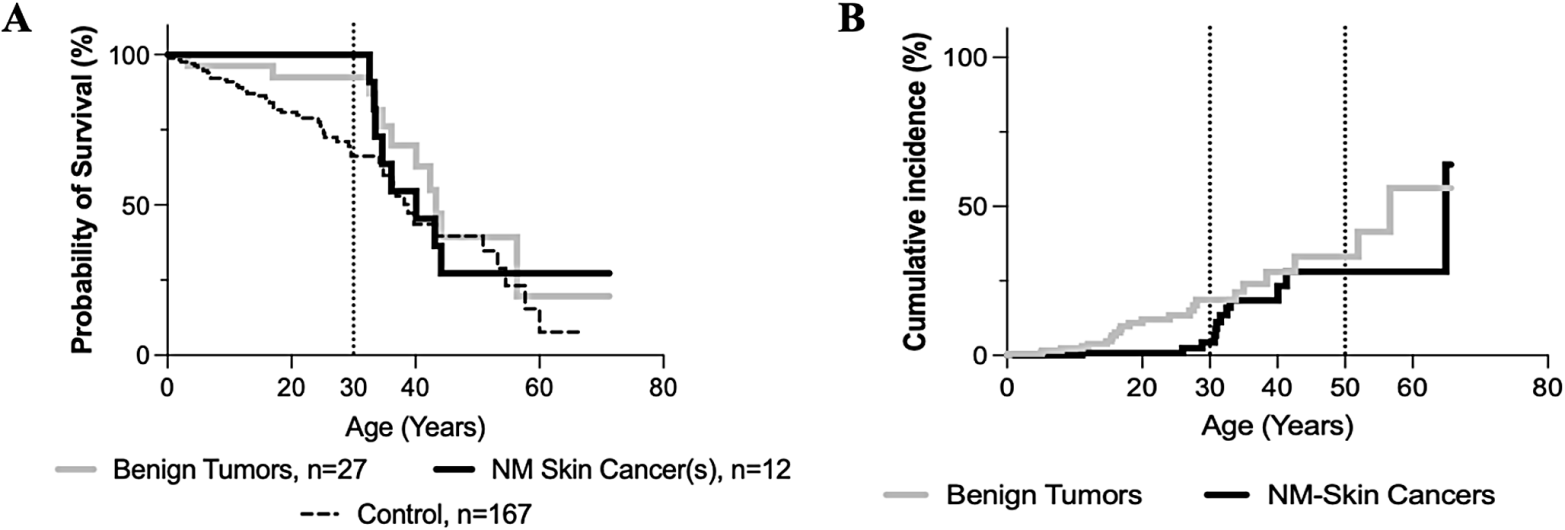
Survival probability and cumulative incidence. NM: non-melanoma (**A**) Survival probability not statistically significant from controls, log rank test *p* = 0.930 (**B**) Cumulative incidence of non-melanoma skin cancer and benign tumors

To assess whether the development of a BT or NMSC indicated disease burden, we examined the cumulative incidence and survival probability of patients with BT and/or NMSC. The cumulative incidence of adverse events in our FA patient cohort, at age 30, was 4.5% for NMSC and 18% for BT. At age 50, the incidence of NMSC and BT increased to 28% and 33% respectively (Fig. 1). To investigate the impact of NMSC and BT on survival probability of FA patients, we compared survival probability of FA patients with an adverse event (NMSC or BT) to the survival probability of FA patients without an adverse event. Our analysis revealed no statistically significant difference in survival probability between the two groups (Log rank test, *p* = 0.0930). Interestingly, the survival probabilities of FA patients with NMSC and BT at age 30 were both 91.5%, a notable contrast to the lower survival probability of 58.5% observed in our control group at the same age (Fig. 1).

## Discussion

Within the cohort of 200 patients with FA, 12 individuals presented with at least one NMSC (SCC or BCC), while 25 patients had at least one BT. Though BT were detected at an earlier age than NMSCs (median 17.5 vs. 31.3, years respectively), patients with FA with skin cancers had them at a younger age compared with the general population. Patients had NMSCs in various locations. Topology information was unavailable for some diagnoses, but many occurred in sun-exposed areas, including the scalp and face. NMSC is known as the most common type of cancer in the general population, but usually does not occur until later in life. For individuals of European ancestry, incidence of NMSC peaks at around 70 years of age and is highly unusual in the teenage years, but were observed at these ages in patients with FA [11]. In a recent study that analyzed statistical associations between clinical parameters in NMSC occurrences, researchers found that the mean diagnostic age was 70.1 years for BCC and 74.2 years for SCC [12]. Patients with FA may be at increased risk of developing an NMSC due to their FA diagnosis (a DNA repair disorder). Additionally, patients with FA may be exposed to radiation or long periods of immunosuppression, which could further increase their likelihood of developing an NMSC.

A prior study on NMSC in individuals with FA yielded results consistent with our findings [13]. In this study, all 8 patients with NMSCs had them at early ages compared to the general population, mirroring our observations. One of their patients had 12 occurrences, while one of our patients had 26. Additionally their youngest patient diagnosed with an NMSC was 10 years old, whereas our youngest patient was 11. These findings are in line with our findings, as well as existing literature, and undercore the importance of proactive monitoring through regular consultations with dermatologists.

A greater proportion of female patients had at least one NMSC event compared to male counterparts. In the general population, several studies found a higher incidence of NMSC in males than in females [14–16]. However, when Evans et al. stratified their analysis based on patient age, they found that in the 10 to 49 year old age cohort, the majority of BCC patients were females, constituting 60.4% of the cases. Conversely, among individuals 50 to 99 years, females accounted for only 36.0% of the BCC cases [14]. This difference in age groups may be due to hormonal effects. Although many women with FA experience early ovarian failure and early menopause, only three women in our cohort had a diagnosis of primary ovarian failure. However, data was unavailable for some women. Taking in consideration the lower survival probability of FA patients compared to the general population, the Evans et al. study could explain the sex distribution found in our NMSC cohort. To further elucidate the potential factors influencing this unusual sex distribution of NMSC in our FA patient group, it is essential to consider unique aspects related to FA, specifically factors that affect sun exposure, health protection behaviors, and lifestyle choices.

The anatomical locations and pathogenesis mechanisms of benign tumors in this group were extremely diverse. This group included both rare and common growths, occurring at various ages, both pre-transplant and following transplant. We did not identify any clear patterns in the distribution of benign tumors. Due to the lack of information in the literature on benign tumors in the general population, we cannot determine whether FA is characterized by an increased risk of benign tumors.

In this case series, one patient (*FANCA*) demonstrated 26 occurrences of NMSC. Most of these instances occurred post-HCT, which was performed at the age of 33, following their diagnosis of FA at 32. This patient also presented with an unspecified immunodeficiency, potentially contributing to the heightened susceptibility to multiple skin cancers. Interestingly, this patient is also heterozygous for two of the seven known BCC risk single nucleotide polymorphisms (SNPs)- rs7538876 (AG) and rs157935 (GT). This patient had the reference allele at rs801114 and rs11170164. No information on SNPs rs401681, rs2151280, and rs78378222 were available for this patient. Collins et al. conducted an investigation on malignancies post-organ transplant which highlighted the increased aggressiveness of NMSC in recipients due to immunosuppression [17]. Although this patient did not undergo organ transplantation specifically, it is possible that a long period of immunosuppression played a role in their many occurrences of NMSC.

One patient experienced all three events studied: SCC, BCC, and benign tumors. The patient had the *FANCA* genotype, and like the patient with 26 NMSC occurrences, received her diagnosis and underwent transplant later in life, at 38 years of age. Prior to the transplant, she was diagnosed with BT and BCC, and subsequently experienced both BCC and SCC post-transplant.

Among the 12 patients diagnosed with SCC or BCC, the majority belonged to the FANCA group (9 patients), with one patient in the FANCC category and two patients with unknown genotypes (Supplemental Fig. 3). Conversely, in the cohort of 25 patients with BT, *FANCA* remained the predominant genotype, accompanied by two *FANCC* cases. However, this group displayed a more diverse genotype distribution, encompassing *FANCI*, *FANCJ* (*BRIP1*), *FANCG*, *FANCD1* (*BRCA2*), and *FANCR* (*RAD51*).

Notably, two patients with the rare *FANCI* genotype presented with BT—one with a colloid cyst of the thyroid and the other with a pineal brain cyst. Additionally, a patient with the rare *FANCR* genotype manifested a BT in the central nervous system, a spinal lipoma. None of these patients underwent HCT, and none experienced SCC or BCC occurrences. All three patients were diagnosed with FA before the age of 6 months.

The single patient with the *FANCD1* genotype presented with both a benign kidney tumor and a parietal lobe tumor in the central nervous system. Additionally, this patient was diagnosed and treated for medulloblastoma at approximately 3 years of age. These occurrences align with existing literature, which indicates that patients with the *FANCD1* genotype tend to develop solid tumors at early ages and have a predisposition to developing brain tumors before 6 years of age [18].

Individuals with either BT or NMSC exhibited a noteworthy increase in survival probability at age 30 compared to the control group, (ns, *p* = 0.0930) (Fig. 1). While the median age at transplant for patients with NMSC was higher than that of patients with BT and controls (30.5, 9.0, 9.1 years, respectively), the median age for patients who developed NMSCs was also greater compared to benign tumor and control groups (33, 17.5, and 15.4 years). Considering these observations, we hypothesize that individuals with FA who develop NMSCs, or BT may have a modifying factor that improved their survival estimates up to age 30, which possibly allowed for the acquisition of DNA damage and subsequent NMSCs. Further investigations into the underlying mechanisms and contributing factors to these tumors and potential modifying factors will be instrumental in determining the nature of these and refining prognostic assessments for individuals with FA.

In 2014, a study characterized the incidence of NMSC in patients enrolled in commercial or Medicare health insurance plans. They found that in United States, NMSC incidence was less than 1% in patients aged 0 to 24, 1% in patients aged 25 to 34, 6% in patients aged 35 to 44 and 17% in patients aged 45 to 54 [14]. For the patients with FA described here, the cumulative incidence of NMSC had a large increase from 4.5% at 30 years to 28% at 50 years (Fig. 1). This jump in incidence emphasizes that age is an important factor in the development of NMSC, and potentially reflects the interplay of genetic and environmental factors in patients with FA. It also highlights the unusual findings of patients with NMSC in their teens/early 20s.

The findings presented in this case series are limited by the relatively small size of the patient cohort, which consisted of 30 individuals with FA and either NMSC or BT. The sample size and ancestry homogeneity of this cohort (86% white), limit the generalizability of our results. Additionally, since most patients with benign tumors were evaluated at the NIH, they were likely evaluated more thoroughly compared to patients for which only medical records review was conducted. This may have increased their likelihood of receiving a benign tumor diagnosis and also limited the generalizability of benign tumor prevalence for this population. However, this cohort of 200 patients with FA is relatively large for this rare disorder. Conducting small case series studies, like the one presented here, becomes imperative to highlight diverse patient profiles and abnormalities within this limited population, even though the results may not be readily generalizable. Another notable limitation is the absence of pathology records for a considerable number of tumors. The lack of these records hinders the comprehensive characterization of the tumors, potentially limiting the depth of insights into their specific nature and behavior. The unavailability of detailed information from pathology reports, including histological subtypes and molecular characteristics, makes it difficult for us to identify patterns in the etiology and progression of the tumors.

In summary, this case study provides a detailed exploration of non-melanoma skin cancers and benign tumors in patients with FA, shedding light on clinical features and factors associated with these events. The findings, consistent with existing literature, underscore the significance of early detection and surveillance of patients with FA, even in cases with milder disease presentations, and highlight the necessity for tailored screening and management strategies for this patient population.

## Electronic supplementary material

Below is the link to the electronic supplementary material.

Supplementary Material 1

## Data Availability

No datasets were generated or analysed during the current study.
