# Correction: Benign tumors and non-melanoma skin cancers in patients with fanconi anemia

**DOI:** 10.1007/s10689-024-00439-3

**Published:** 2025-01-18

**Authors:** Aura Enache, Bia Sajjad, Burak Altintas, Neelam Giri, Lisa J. McReynolds, Edward W. Cowen

**Affiliations:** Division of Cancer Epidemiology and Genetics, Clinical Genetics Branch, National Cancer Institute, 9609 Medical Center Drive 6E434, Bethesda, MD 20892 USA

**Correction: Familial Cancer**

10.1007/s10689-024-00410-2

In the original version of this article, one of the co-authors name “Edward W. Cowen” were not included.

The original article has been corrected.

