# Supplementary material for: Benign tumors and non-melanoma skin cancers in patients with Fanconi anemia
Source: Fam Cancer. 2024 Jun 21;23(4):583–90. doi: 10.1007/s10689-024-00410-2 (PMC11512875; doi:10.1007/s10689-024-00410-2)
Supplement: Supplementary file 1 — Supplementary Material 1 [file 10689_2024_410_MOESM1_ESM.pdf]

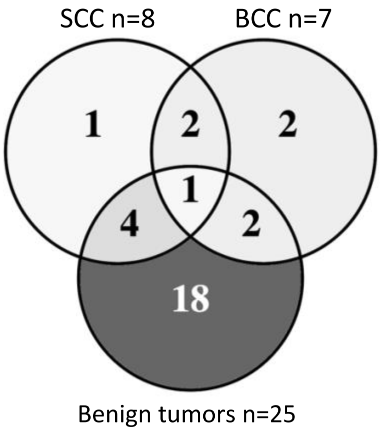

Supplemental figure 1. Adverse event distribution

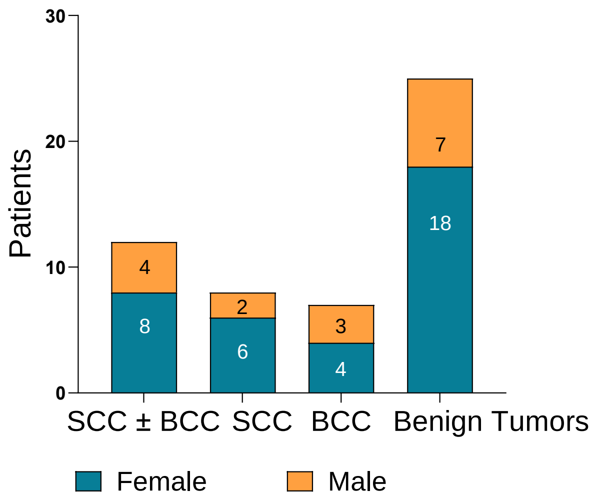

Supplemental figure 2. Sex distribution

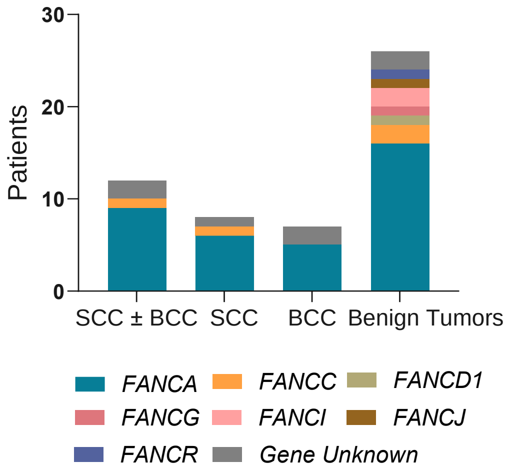

Supplemental figure 3. FA gene association
